# Spatio-Temporal Study of Galactolipid Biosynthesis in Duckweed Using Mass Spectrometry Imaging and *in vivo* Isotope Labeling

**DOI:** 10.1093/pcp/pcae032

**Published:** 2024-03-28

**Authors:** Vy T Tat, Young Jin Lee

**Affiliations:** Department of Chemistry, Iowa State University, 2415 Osborn Drive, Ames, IA 50011, USA; Department of Chemistry, Iowa State University, 2415 Osborn Drive, Ames, IA 50011, USA

**Keywords:** Digalactosyldiacylglycerol, Galactolipids, *in vivo* isotope labeling, *Lemna minor*, Mass spectrometry imaging, Monogalactosyldiacylglycerol

## Abstract

Isotope labeling coupled with mass spectrometry imaging (MSI) presents a potent strategy for elucidating the dynamics of metabolism at cellular resolution, yet its application to plant systems is scarce. It has the potential to reveal the spatio-temporal dynamics of lipid biosynthesis during plant development. In this study, we explore its application to galactolipid biosynthesis of an aquatic plant, *Lemna minor*, with D_2_O labeling. Specifically, matrix-assisted laser desorption/ionization-MSI data of two major galactolipids in *L. minor*, monogalactosyldiacylglycerol and digalactosyldiacylglycerol, were studied after growing in 50% D_2_O media over a 15-day time period. When they were partially labeled after 5 d, three distinct binomial isotopologue distributions were observed corresponding to the labeling of partial structural moieties: galactose only, galactose and a fatty acyl chain and the entire molecule. The temporal change in the relative abundance of these distributions follows the expected linear pathway of galactolipid biosynthesis. Notably, their mass spectrometry images revealed the localization of each isotopologue group to the old parent frond, the intermediate tissues and the newly grown daughter fronds. Besides, two additional labeling experiments, (i) ^13^CO_2_ labeling and (ii) backward labeling of completely 50% D_2_O-labeled *L. minor* in H_2_O media, confirm the observations in forward labeling. Furthermore, these experiments unveiled hidden isotopologue distributions indicative of membrane lipid restructuring. This study suggests the potential of isotope labeling using MSI to provide spatio-temporal details in lipid biosynthesis in plant development.

## Introduction

Over the last two decades, metabolomics has elucidated the metabolic responses of plants to various perturbations and provided a deeper understanding of metabolic networks (Alseekh and Fernie 2018). Furthermore, many gene functions were able to be annotated through the metabolomics of knock-out mutants, quantitative trait loci mapping and genome-wide association studies. Most of these studies are based on chromatographic separation followed by mass spectrometry (MS) analysis of whole tissue extracts and are unable to distinguish metabolites from different cell types. Additionally, metabolite concentrations alone do not directly probe metabolic activities (Jang et al. 2018). Two new strategies have recently emerged to enlighten the spatial or temporal dynamics of plant metabolism, which was not possible with traditional metabolomics tools. One is isotope labeling and tracing labeled metabolites, which was used to annotate metabolites (Huang et al. 2014), elucidate pathway structures and analyze metabolite flux (Allen et al. 2015, Wang et al. 2018). The other is MS imaging (MSI), which visualizes each metabolite directly on tissues at single-cell resolution (Lee et al. 2012). Both techniques rely on MS when a stable isotope is used, but the combination of the two, namely, MSI with *in vivo* isotope labeling which we call MSI*i*, has been rarely applied to plant systems.

There are two notable studies of MSI*i* in plants. We have previously visualized free amino acids in maize root sections grown in ^15^NH_4_Cl medium to differentiate external nitrogen from those transported from seeds. This study demonstrated the genotypic difference in amino acid localization and their indifference to the nitrogen source (O’Neill and Lee 2020). Visualization of ^13^C-labeled phosphatidylcholine (PC) species in developing Brassica seeds (camelina and pennycress) was achieved by Romsdahl and coworkers by feeding siliques with ^13^C-glucose (Romsdahl et al. 2021). They revealed a greater ^13^C-labeling in cotyledons than in embryonic axis and in PC species with saturated and longer acyl chains. Other MSI*i* studies in plants include ^15^N-labeling of *Catharanthus roseus* to assist nitrogen-containing metabolite identifications (Nakabayashi et al. 2020) and the labeling of lemna and tomato with D_4_- and ^13^C_9_-tyrosine to study the tyrosine-derived metabolic network (Feldberg et al. 2018).

To further explore the potential of MSI*i*, here, we adopt D_2_O labeling to study the galactolipid biosynthesis in *Lemna minor*. Unlike other isotope labeling, D_2_O is a global labeling agent in plants as all hydrogen atoms are originated from water and ‘fixed’ during photosynthesis (Nett et al. 2018). Heavy water (D_2_O) labeling is previously used in plants to produce deuterated biomass in annual ryegrass (Evans et al. 2014) and *L. minor* (Evans et al. 2019), to investigate *de novo* synthesis of volatile terpenes in *Achyranthes bidentata* (Tamogami et al. 2013), to measure turnover rates of *Arabidopsis* proteins (Yang et al. 2010) and to measure the biosynthetic rate of the cytokinin class of plant hormones in *Arabidopsis thaliana* (Astot et al. 2000) and various natural products in medicinal plants (Nett et al. 2018). However, it has not been used in MSI*i* of plants, although used in mouse to visualize the rapidly growing tumor region (Louie et al. 2013).

As an aqua plant, duckweed, *Lemna*, is very attractive for MSI*i* using D_2_O. High D_2_O concentration is toxic to all organisms, but *Lemna* species can grow in up to 65% D_2_O with supplementation (Cope et al. 1965). It can avoid problems associated with D_2_O, such as germination rupture or root elongation, since it grows by budding from fronds (Evans and Shah 2015). Evans and coworkers have successfully cultivated *L. minor* in 50–60% D_2_O and studied the morphological and biochemical properties of D-labeled plants (Evans et al. 2019). Fatty acid (FA) and protein syntheses are commonly studied with D_2_O as an isotopic tracer to measure pathway activities (Jang et al. 2018), but they often require saponification or hydrolysis, which is not adequate for MSI with direct sampling on tissue surfaces. Instead, galactolipids, the most abundant chloroplast membrane lipids, are chosen in this study because of their high abundance in matrix-assisted laser desorption/ionization (MALDI)-MS, a technical platform used in this study for MSI. A partial D_2_O labeling (e.g. 50%) leads to low signals for each isotopologue due to the binomial distribution of H- vs D-labeling; as a result, it was essential to study highly abundant lipids in this first application of D_2_O labeling for MSI*i*.

Chloroplasts are the home of thylakoids where photosynthesis and many other essential biosynthesis processes occur in plants. Two galactolipids are the major components of thylakoid membrane lipids, monogalactosyldiacylglycerol (MGDG) and digalactosyldiacylglycerol (DGDG). Most steps in galactolipid biosynthesis are localized to chloroplasts, including photosynthesis (glucose synthesis), FA synthesis and the final step of galactosylation. However, the utilization of FAs is different between prokaryotic and eukaryotic pathways. In the prokaryotic pathway, *de novo*–synthesized 16:0 and 18:1 FAs are directly used to synthesize galactolipids within the plastid envelope. In the eukaryotic pathway, they are exported to ER for eukaryotic phospholipid synthesis, such as PC and phosphatidylethanolamine. Some portion of diacylglycerol (DAG) precursors, e.g. PC or phosphatidic acid (PA), are returned to the chloroplast to produce galactolipids (Hölzl and Dörmann 2019). While the biosynthetic pathways are well-established, there has been no study on the spatio-temporal dynamics during leaf development. *Lemna* proves to be an excellent system to investigate this aspect with MSI*i*, given that its budding process enables the observation of the spatio-temporal advancements in new lipid biosynthesis.

## Results

### MALDI-MS of *L. minor* grown in 50% D_2_O medium

**Fig. 1:**
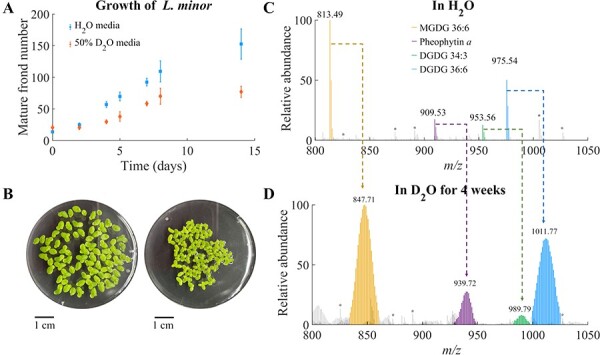
Growing *L. minor* in the D_2_O medium. (A) The growth rate of *L. minor* in the H_2_O vs 50% D_2_O 0.5× SH medium. (B) The images of *L. minor* after growing in H_2_O (left) vs 50% D_2_O medium (right) for 4 weeks. The averaged MALDI mass spectra of *L. minor* grown in (C) H_2_O medium vs (D) 50% D_2_O medium. All galactolipids and pheophytin *a* are detected as potassium adducts. There are some background peaks (*) coming from the non-tissue area.

In the current simple experimental set-up, we opted to grow duckweeds in a Petri dish with 0.5× Schenk and Hildebrandt (SH) medium on a laboratory bench. As expected, room humidity affected the D_2_O concentration overtime due to water vapor exchange, but it was minimal when the humidity was low, which will be further discussed in section ‘Limitations of this study’. Unlike the previous work of Evans et al. (2019), supplementation with 0.5% glucose was excluded to avoid fungal growth and its effect on lipid biosynthesis. Initially, we added glucose to both H_2_O and 50% D_2_O media and sterilized plants using multiple methods, but fungal growth was not avoidable in this set-up. As shown in **Fig. 1A** and **Supporting Video S1**, the growth of *L. minor* was about twice slower in 50% D_2_O medium than in H_2_O. Starting with mature fronds of the same size, the second daughter fronds appeared after 3 d in 50% D_2_O media, which were 2 d later than those grown in H_2_O. At the end of the week, the second daughter fronds in 50% D_2_O reached about a quarter size of mature frond, whereas those in the H_2_O media were slightly smaller than the mature one. Additionally, the fronds grown in 50% D_2_O were thicker and smaller (**Fig. 1B**). Lastly, roots in 50% D_2_O medium were hardly seen, while they were about 1 mm in the control condition. Nevertheless, *L. minor* looked healthy otherwise and could grow in 50% D_2_O indefinitely with regular medium change. Despite some differences, growing *L. minor* in D_2_O medium seems to have relatively minor adverse effects from D_2_O stress and could be used to monitor their D-labeling in lipid biosynthesis.

To obtain the MALDI-MSI data of the thylakoid membrane lipids across the lateral dimension of duckweed fronds, a fracturing method is employed to horizontally split *L. minor* fronds into two halves (See the Methods section for the details). As we have previously demonstrated (Klein et al. 2015), the exposed internal mesophyll layers produce mostly hydrophobic lipid profiles when interrogated by MALDI-MS. MALDI-MS spectra were acquired throughout the fractured plant, pixel by pixel, with each *x*, *y* information for later imaging analysis. **Fig. 1C and D** compare the averaged MALDI-MS spectra of the *L. minor* grown in H_2_O and D_2_O media for 4 weeks, a typical lifespan of *L. minor* under laboratory conditions (Ashby et al. 1949), after being prepared by the fracturing method. The MALDI-MS spectrum under the H_2_O condition (**Fig. 1C**) was dominated by galactolipids, MGDG 36:6, DGDG 36:6, DGDG 34:3 and pheophytin *a*. The abundance of MGDG 36:6 and DGDG 36:6 was significantly greater than that of DGDG 34:3, consistent with the fact that *L. minor* was reported as 18:3 plant with C_18_ FA at the *sn*-2 position through the eukaryotic pathway (Mongrand et al. 1998). In the MALDI-MS condition, chlorophyll *a* readily loses non-covalently bound Mg^2+^ and is detected as pheophytin *a*. After growing in the 50% D_2_O medium for 4 weeks, the galactolipids and pheophytin *a* were significantly labeled by deuterium as shown in **Fig. 1D**. Notably, no unlabeled, monoisotopic peaks were left in the mass spectrum, because parent fronds died and were separated out. The D-labeling efficiency of galactolipids seems to be close to 100% considering the mass difference between the monoisotope in H_2_O medium and the most abundant isotope in the 50% D_2_O medium. For example, MGDG 36:6 has the mass difference of ∼34.2 Da corresponding to ∼97% D-labeling efficiency out of 50% (D_2_O concentration) of its 70 carbon-bound hydrogens (C–H): 34.2 Da/(50% × 70 × 1.006277 Da) where 1.006277 Da corresponds to the mass difference between H and D atoms.

### Temporal change of galactolipid isotopologues with D_2_O labeling

**Fig. 2:**
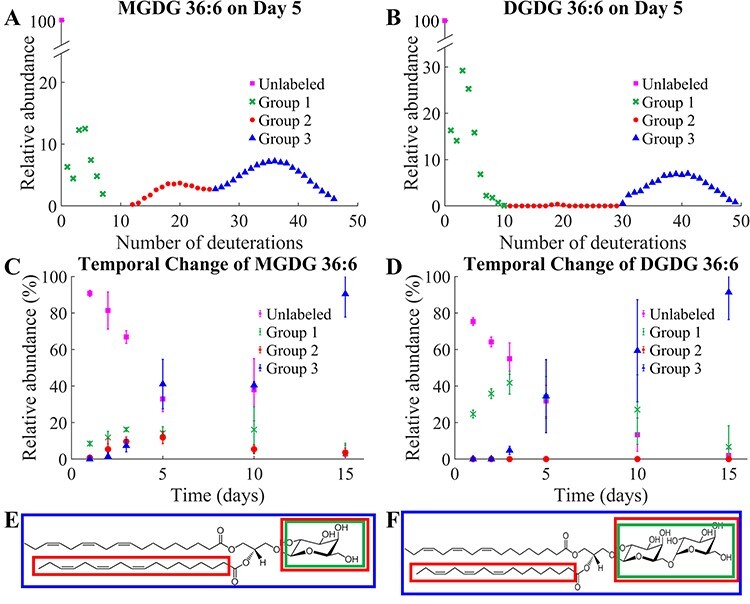
D_2_O-labeled galactolipids and their temporal changes. D-Labeling isotopologue distribution of (A) MGDG 36:6 and (B) DGDG 36:6 after growing *L. minor* for 5 d in the 50% D_2_O medium. ^13^C- or other natural isotope abundance is subtracted as described in **Supporting Methods**. The temporal change of each D-labeling group over time for (C) MGDG 36:6 and (D) DGDG 36:6. Three replicates of *L. minor* were used for each time point. The structures of (E) MGDG 36:6 and (F) DGDG 36:6 indicate each D-labeled structural moiety.

MALDI-MS data of *L. minor* were obtained after various time points grown in the 50% D_2_O medium to monitor the temporal change of D-labeling in these lipids. **Fig. 2A, B** shows the isotopologue distributions of MGDG 36:6 and DGDG 36:6 on day 5. Because of insufficient mass resolution to separate ^13^C- or other natural isotopes from D-labeled peaks, natural isotope abundances were subtracted as described in **Supporting Methods** and **Fig. S1** to obtain D-labeling isotopologue distributions. There might be some subtraction errors in this process due to the isotope abundance measurement error in mass spectrometers. MGDG 36:6 exhibited three binomial isotopologue distributions centered around the average D-labeling of 3.5, 18 and 35, which were referred to groups 1, 2 and 3, respectively (**Fig. 2A**). The three distinct D-labeling isotopologue groups were attributed to the D-labeling of different structural moieties in MGDG (**Fig. 2A, E**). Assuming 100% D-labeling efficiency, group 1 corresponds to the D-labeling of the galactosyl group; when half of the seven carbon-bound hydrogen atoms (7 C–H) in the galactose moiety are deuterium-labeled, the count amounts to 3.5. Group 2 corresponds to the D-labeling of the galactosyl group and a fatty acyl chain (FA); half of 36 C–H in FA 18:3 and galactose is 18. Group 3 corresponds to the D-labeling of the whole molecule; half of 70 C–H in MGDG 36:6 is 35. **Fig. S2** shows the binomial distributions simulated for groups 1, 2 and 3, assuming *p* (D_2_O concentration) of 0.5 and *n* (number of C–H) of 7, 36 and 70, respectively. They are very closely matching with the experimental isotopologue distributions, especially at the peak positions, although experimental data are slightly broader for groups 2 and 3.

DGDG had a similar labeling trend to MGDG but mostly with two groups, groups 1 and 3 (**Fig. 2B**). Group 2 was barely present in a very low abundance, and it seemed real according to MALDI imaging (see the next section ‘Spatial distribution of galactolipid isotopologues with D_2_O labeling’). It could be clearly seen in electrospray ionization (ESI)-MS analysis in **Fig. S3** when multiple plants are combined for the lipid extraction. In a closer look, the D-labeling of group 1 was extended to at least 10 deuterations for DGDG 36:6, which was attributed to the second galactosyl group labeling with the average D-labeling of 7, half of 14 C–H in digalactose. The contribution from the second galactose labeling increased over time and became clearer in later days as shown in **Fig. S4**, but the first galactose labeling was still dominant even on day 15.

The temporal change of three D-labeling groups is shown in **Fig. 2C** for MGDG 36:6, along with the unlabeled monoisotope. Group 1 emerged rapidly on day 1, increased slightly until day 3 and then remained at a similar level until day 10. Group 2 showed up on day 2, increased slowly until day 5 and then decreased. Group 3 did not clearly appear until day 3 but rapidly increased and became dominant by day 15. It should be noted that unlabeled galactolipids were almost gone on day 15 because the parent frond was separated from the daughter fronds by then. DGDG 36:6 showed similar temporal changes to those of MGDG for groups 1 and 3 (**Fig. 2D**) but the relative abundance of group 1 is more than twice that of MGDG group 1. Unlike galactolipids, pheophytin *a* had only a single isotopologue distribution corresponding to the D-labeling of the entire molecule over the 15-day period (**Fig. S5**).

### Spatial distribution of galactolipid isotopologues with D_2_O labeling

**Fig. 3:**
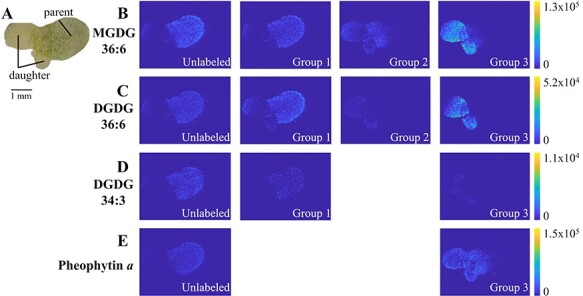
MALDI-MS images of *L. minor* after growing in 50% D_2_O for 5 d. (A) Optical image of the top half-fractured and MS images of each D-labeling group for (B) MGDG 36:6, (C) DGDG 36:6, (D) DGDG 34:3 and (E) pheophytin *a*. Group 1 does not contain M1 and M2 to avoid the contribution from the natural isotope of monoisotope.

From the MALDI-MS data obtained across the entire tissues, MS images can be constructed for each *m*/*z* species using *x*, *y* information of each mass spectrum. **Fig. 3** shows the MS images of three major galactolipids and pheophytin *a* for a duckweed grown in 50% D_2_O for 5 d, the same data used in **Fig. 2A, B**. The group images were obtained by combining all the deuterated peaks for the same group, and the MS images were essentially identical within the same group (**Supporting Video S2**). Some contamination of natural isotopes was unavoidable in the imaging construction, especially ^13^C_1_ and ^13^C_2_ contribution to D_1_- and D_2_-labeling in group 1; however, its contribution is mostly ignorable in the overall images.

As can be seen in the optical image of a fractured half (**Fig. 3A**), a duckweed plant typically has three fronds, one parent and two daughter fronds. The unlabeled monoisotope peaks were present almost exclusively in the parent frond, which was grown in the H_2_O medium before transferring into the D_2_O medium (**Fig. 3B–D**). Group 1 of all galactolipids was co-localized to the unlabeled monoisotope, whereas group 3, the labeling of the whole molecule, was present only in the daughter fronds, newly grown tissues in the D_2_O medium. Lastly, group 2 of MGDG showed an intermediate behavior between group 1 and 3 images, present in the newer part of the parent frond (i.e. near the base) or the older part of daughter fronds (i.e. near the margin). Interestingly, pheophytin *a* has only group 3 that appeared not only in the new daughter fronds but also in intermediate tissue regions including the base of the parent frond (**Fig. 3E**). This suggests that chlorophyll *a* was rapidly synthesized with full D-labeling even in the intermediate tissues, which agrees with the fact that pheophytin *a* is fully D-labeled even on day 2 (**Fig. S5**).

This observation in MS imaging was further supported by the ESI-MS of lipid extracts from *L. minor* grown in the 50% D_2_O medium for 5 d (**Fig. S3**). For the lipids extracted from the parent fronds, the isotopologue distribution was dominated not only by the unlabeled monoisotope and group 1 but also by a little amount of group 2, whereas the lipids extracted from daughter fronds were mostly group 3 and a small amount of groups 1 and 2. This trend is expected from MALDI-MS images in **Fig. 3**.

### Preliminary ^13^CO_2_-labeling experiment

We have shown that D_2_O labeling could successfully capture some intermediates of galactolipid biosynthesis and their spatio-temporal behaviors. There is a major concern that the observation of three isotopologue groups is a result of D_2_O-induced artifact. To test whether the observed spatio-temporal behavior is consistent without D_2_O stress, we performed a simple experiment of ^13^CO_2_-labeling by growing duckweeds in a small chamber with ^13^CO_2_. It is described in detail in the Experimental section, but in short, duckweeds were grown in a small beaker with 0.5× SH medium inside a sealed large flask **(Fig. S6)**. The ambient air inside the flask was flushed out with CO_2_-free air, and ^13^CO_2_ was produced using Ba^13^CO_3_. As there was a minor leak in the system, air flush and new ^13^CO_2_ production were repeated every 3 h during the daytime, with the first cycle of a day coinciding with the activation of light emitting diode (LED) lights, the onset of photosynthesis. The ^13^CO_2_ concentration was estimated to be around 1,500 ppm when freshly produced. It was a much higher CO_2_ concentration than in ambient air but on purpose to minimize ^12^CO_2_-labeling due to the leak.

**Fig. 4:**
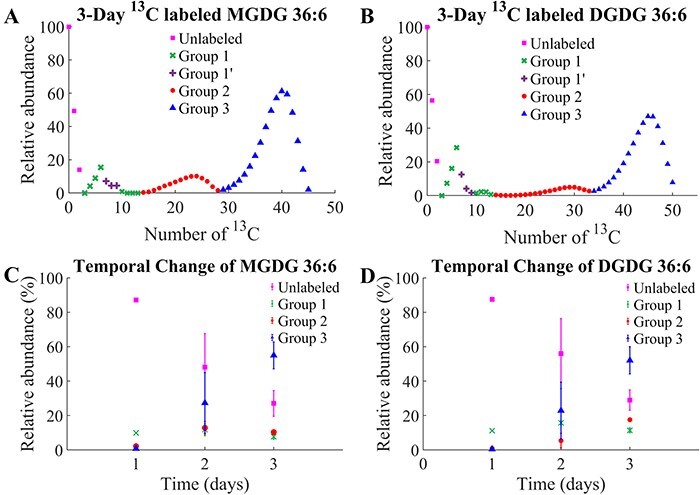
^13^CO_2_-labeled galactolipids and their temporal changes. Isotopologue distribution of (A) MGDG 36:6 and (B) DGDG 36:6 after growing *L. minor* for 3 d in ^13^CO_2_. Natural isotope abundance was not subtracted to avoid confusion. The temporal change of each ^13^C-labeling groups over time for (C) MGDG 36:6 and (D) DGDG 36:6. Group 1ʹ is combined with group 1 in the temporal change as it cannot be separated in DGDG, and they both are present in the parent frond. Three replicates of *L. minor* were used for each time point.

Unlike D_2_O labeling, no visual difference was apparent between control and ^13^C-labeled fronds. Three replicate plants each were subjected to MALDI-MSI analysis after growing in ^13^CO_2_ for 1–3 d. The ^13^C-isotopologue distributions of MGDG 36:6 and DGDG 36:6 on day 3 (**Fig. 4A, B**) have three isotopologue groups similar to D_2_O labeling. Unlike symmetric distributions in D-labeling, isotopologue distributions in ^13^C-labeling were skewed toward a higher isotope due to a much higher ^13^CO_2_ concentration than ^12^CO_2_. In addition to galactose labeling of group 1, glycerol backbone labeling was observed and marked as group 1ʹ, which is further explained in the Discussion section. The temporal change of the three isotopologue groups (**Fig. 4C, D**) also shows a similar trend as in D-labeling with unlabeled decreasing and group 3 increasing over time, while groups 1 and 2 increased fast initially and then stabilized. A similar trend is expected to continue in a longer experiment although we did not perform longer than 3 d due to the experimental difficulty in the current set-up. The decrease in unlabeled and the increase in fully labeled were much faster than in D-labeling. For example, group 2 vs group 3 ratio of MGDG 36:6 in day 2 ^13^C-labeling (**Fig. 4C**) is already comparable to that on day 5 of D-labeling (**Fig. 2C**). It was mostly attributed not only to a faster growth of *L. minor* in the H_2_O medium (**Fig. 1A**) but also partially to a much higher ^13^CO_2_ concentration.

**Fig. 5:**
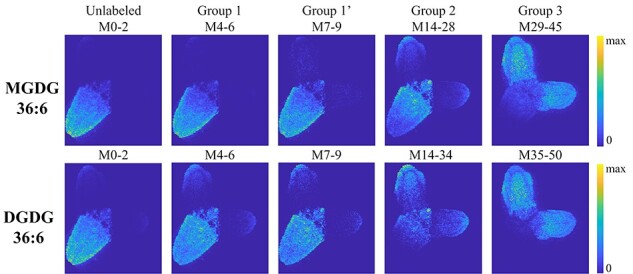
MALDI-MS images of *L. minor* after growing in the ^13^C-chamber for 3 d. The maximum scale is arbitrarily adjusted for each image.

MALDI-MS images are shown in **Fig. 5** for each ^13^C-isotoplogue group of MGDG 36:6 and DGDG 36:6. Overall, they show similar images with D-labeling in **Fig. 3**. Unlabeled galactolipids were present mostly in the parent frond as well as groups 1 and 1ʹ, while group 3 was present almost exclusively in daughter fronds. Group 2 showed an intermediate behavior between groups 1 and 3 for both galactolipids, but a little more localized to the parent frond for MGDG and to the daughter fronds for DGDG. It is consistent between ^13^C- and D-labeling that one and two FA(s) labeling represented by groups 2 and 3, respectively, have appearance in newer tissues, distinct from old tissues at the time of starting the labeling experiments.

### Backward labeling

**Fig. 6:**
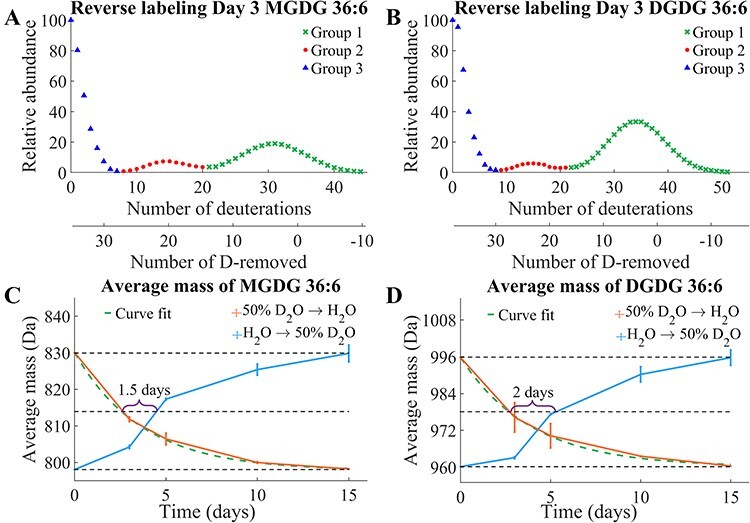
Backward labeling of *L. minor*. Isotopologue distribution of (A) MGDG 36:6 and (B) DGDG 36:6 3 d after moving *L. minor* grown in 50% D_2_O for 3 months to the H_2_O medium. A secondary *x*-scale is shown in the reverse order as the number of D-removed, defined as the average number of full deuteration (34.5 for MGDG 36:6 and 38 for DGDG 36:6) subtracted by the number of deuterations. The natural isotope abundance was not subtracted to avoid confusion and calculation error. Temporal change in the average mass of (C) MGDG 36:6 and (D) DGDG 36:6. These data were obtained by TLC-ESI-MS of lipid extract from three replicates of 15–20 plants at each time point. In forward labeling, the average mass of MGDG 36:6 and DGDG 36:6 was reached 1.5–2 d later than in backward labeling. For the backward labeling, average mass change is fitted with an exponential decay function at *R*^2^ > 0.99.

To further support the observed isotopologue distributions, we have performed another supporting experiment by performing backward labeling. In this experiment, we have established that *L. minor* adapted to D_2_O stress via multiple generations of culture in the 50% D_2_O medium for 3 months. They were then moved back to H_2_O media and monitored the change in their isotopologue patterns over a 15-day period. In this case, we expect that the labeling would occur reversely, in which more unlabeled moieties and molecules would be produced over time. Isotopologue distributions on day 3 are shown in **Fig. 6A, B** for MGDG 36:6 and DGDG 36:6, respectively. Fully labeled *L. minor* grown in 50% D_2_O for 3 months had only group 3 isotopologue distributions with the average number of deuterations of ∼34.5 and ∼38 for MGDG 36:6 and DGDG 36:6, respectively (not shown). To make it easier to understand the backward labeling data, a secondary *x*-scale is shown in reverse order as the number of D-removed. The number of D-removed is defined here as the average full deuteration (34.5 for MGDG 36:6 and 38 for DGDG 36:6) subtracted by the number of deuterations. As expected, three groups of isotopologue distributions are observed similar to forward labeling (i.e. D_2_O labeling) corresponding to D-removal of galactose (group 1), intermediate D-removal (group 2) and full D-removal (group 3). It should be noted that although we call it ‘D-removal’ for the convenience of explanation, it is actually H-labeling in new biosynthesis that dominates D-labeling in the total population.

The major differences between the forward (**Fig. 2**) and backward labeling (**Fig. 6**) are that (i) the isotopologue distribution was very broad in group 1 and became narrower with D-removal (or H-labeled) in group 3 and (ii) D-removal (or H-labeling) was much faster than D-labeling that group 3 was already ∼40% of total on day 3 for both MGDG 36:6 and DGDG 36:6, a similar level on day 5 of D-labeling. The labeling time difference could be attributed to the D_2_O-induced stress and corresponding slower growth in D_2_O (**Fig. 1A**). As another and more quantitative measure of D_2_O-stress induced growth delay, we calculated the average mass from the entire isotopologue and traced their changes over the 15-day period as shown in **Fig. 6C**, **D** for MGDG 36:6 and DGDG 36:6, respectively. As expected, the average mass increased over time for forward labeling and decreased for backward labeling. Assuming that half-way change in the average mass represents the overall growth rate or total D- (or H-) labeling rate, there is about 1.5∼2 d of delay in forward labeling compared to backward labeling, presumably due to D_2_O-induced stress. Interestingly, the trend of backward labeling has a simple gradual decrease that can be fitted with an exponential decay function at *R*^2^ of >0.99; however, the forward labeling shows a S-curve trend, suggesting that the 2-day delay might be mostly due to the initial adjustment time to the new D_2_O stress environment.

## Discussion

### Spatio-temporal pattern of D-labeled isotopologue groups follows a linear pathway of galactolipid biosynthesis

**Fig. 7:**
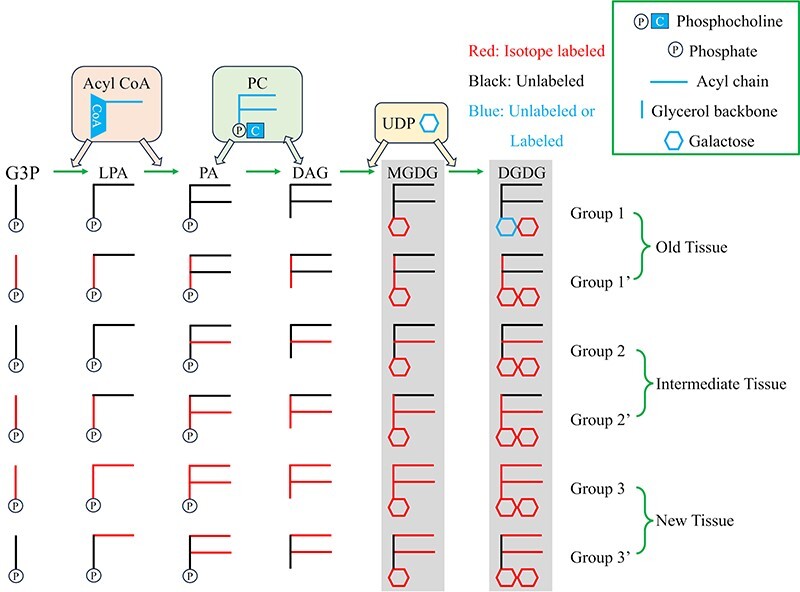
Simplified diagram for galactolipid biosynthesis with D_2_O labeling. Abbreviations: CoA, coenzyme A; G3P, glycerol-3-phosphate.

In this work, we have successfully demonstrated that *in vivo* D_2_O-labeling can be used to capture metabolite intermediates in galactolipid biosynthesis and monitor their temporal changes and spatial distributions. The spatio-temporal pattern of three D-labeled isotopologue groups (groups 1, 2 and 3 in **Figs. 2** and **3**) can be explained by galactolipid biosynthesis (Ohlrogge and Browse 1995) illustrated in **Fig. 7** with the isotope labeled building block. The major difference among the isotopologue groups is the number of fatty acyl chains labeled and their localizations: groups 1, 2 and 3 have no, one and two fatty acyl chains labeled, respectively, with the localization in old, intermediate and new tissues. The interpretation of groups 1, 2 and 3 is straightforward as they are in reverse order in the biosynthesis. Namely, group 1 labeling occurs in the old tissues via the last step of labeled galactose attachment to the unlabeled precursors. Group 2 labeling starts with unlabeled lysophosphatidic acid (LPA) as a precursor that was available in the intermediate tissues by adding a deuterated FA and galactose(s). Lastly, group 3 is observed only in new tissues that were grown after being moved to D_2_O medium as the entire molecule is D-labeled. Their temporal appearances are also in the order of groups 1, 2 and 3, as expected. In a closer look, there is some partial overlap in the localization between groups 1 and 2 as well as between groups 2 and 3 (**Figs. 3**, **5**). This suggests that the boundary of some intermediate tissues is not clear.

The observation of three distinct major isotopologue groups, referred to as groups 1, 2 and 3, in *in vivo* isotope labeling of galactolipids was possible as a result of (i) the time scale difference in biosynthesis of their building blocks, (ii) the high enough abundance of precursors and (iii) sufficient separation in mass dimension. First, sugars, such as galactose, are rapidly synthesized and their attachment to DAG or MGDG to produce MGDG group 1 or DGDG group 1 appears just 1 d after moving to the D_2_O medium. Meanwhile, it took one and two more days for the obvious presence of groups 2 and 3, respectively. If the overall biosynthesis occurred rapidly without the distinct time scale difference between each step, only group 3 would have been observed as in pheophytin *a* (**Fig. 3**, **Fig. S5**). Second, each isotopologue group would not have been observed above the detection limit if there were insufficient amounts of precursors. For example, group 1 of MGDG is composed of unlabeled DAG and deuterated galactose (dGal), in the form of ‘DAG-dGal’ (**Fig. 7**). Hence, the presence of a large pool of unlabeled DAG precursors (e.g. unlabeled PC or PA) in old tissues would be necessary for the observation of MGDG group 1. Similarly, the observation of group 2 suggests the presence of a relatively high abundance of unlabeled LPA in intermediate tissues so that D-labeled FA and D-labeled galactose can be attached to produce MGDG or DGDG group 2. Finally, the observation of three isotopologue groups was not possible without a clear mass difference between them. We used almost the maximum D_2_O concentration possible for *L. minor*; yet, a broad binomial distribution is unavoidable in this study. Fortunately, many C–H in fatty acyl chains (29 for FA 18:3) was sufficient to separate at least three isotopologue groups with no (group 1), one (group 2) and both (group 3) fatty acyl chains labeled.

### Partial glycerol labeling is present in all isotopologue groups

In addition to groups 1, 2, and 3 observed in D_2_O labeling, ^13^C-labeling and backward labeling uncovered other isotopologue groups named groups 1ʹ, 2ʹ and 3ʹ that are hidden within the wide isotopologue distributions of D-labeling. They contain additional building blocks that are not in the order of galactolipid biosynthesis, suggesting the mixed presence of labeled and unlabeled building blocks. First, ^13^C-labeling revealed the presence of group 1ʹ corresponding to the labeling of glycerol in addition to galactose labeling. The three ^13^C-isotopologue distributions for MGDG 36:6 in **Fig. 4A** could be explained by the simulated binomial distributions in **Fig. S7A** using *p* (^13^CO_2_ concentration) of 0.9 and *n* (carbon number) of 6 (galactose), 24 (galactose + FA 18:3) and 45 (entire molecule) for groups 1, 2 and 3, respectively. Experimental isotopologue distributions were broader for groups 2 and 3 than in the simulation, most likely because the ^13^CO_2_ vs ^12^CO_2_ concentration was continuously changing over time due to the leak. An interesting observation was a distinct distribution of ^13^C_7_-^13^C_9_ isotopologues (or M7–M9) in **Fig. 4A**, named as group 1ʹ because of its co-localization with group 1 (**Fig. 5**). The simulation in **Fig. S7A** with *n* = 9 (galactose + glycerol) can explain for the presence of group 1ʹ (M7–M9 isotopologue). Galactose and glycerol backbone labeling was not observed in D-labeling partially because of the broad nature of D-labeling isotopologue distributions. Group 1ʹ was also observed for DGDG 36:6 although it overlapped with ^13^C-labeling of the second galactose (**Fig. 4B**). Unlike the simulation, ^13^C_7_-labeling has a higher abundance than ^13^C_8_ or ^13^C_9_-labeling, suggesting that there might be some contribution from the recycling of unlabeled moieties. The presence of group 1ʹ in old tissue does not follow the linear pathway of galactolipid biosynthesis as illustrated in **Fig. 7**, namely, unlabeled FAs are attached to the newly synthesized ^13^C-glycerol backbone. This may suggest that membrane restructuring is continuously occurring in old tissues but recycling unlabeled FAs (Yu et al. 2021).

Second, ^13^C-labeling and the backward labeling revealed the presence of group 2ʹ, another intermediate labeling with not only one FA and galactose but also glycerol backbone labeled. In a closer look of the ^13^C-isotopologue profile (**Fig. 4**), group 2 peak positions of 24 and 30 for MGDG 36:6 and DGDG 36:6, respectively, were slightly higher than 22 and 28 in the simulated peak positions of group 2 (**Fig. S7**). They were rather closer to 25 and 30 in the simulated peak positions of group 2ʹ calculated with *n* = 27 (galactose + FA 18:3 + glycerol) and 33 (two galactose + FA 18:3 + glycerol), respectively, corresponding to glycerol labeling in addition to group 2. It may suggest that group 2 also had some contribution of the ^13^C-labeled glycerol backbone although they cannot be separated from group 2 due to the wide isotopologue distributions. Another evidence of group 2ʹ can be found in the backward labeling data (**Fig. 6**). Group 2 was shifted by ∼19.7 D-removal (or H-labeling) for MGDG 36:6 and ∼22.6 D-removal (or H-labeling) for DGDG 36:6 from the fully D-labeled initial positions. These shifts were larger than the labeling of galactose and a fatty acyl chain in forward labeling, 18 and 21.5 for MGDG and DGDG, respectively (half of 36 C–H and 43 C–H). Similar to ^13^C-labeling, this is attributed to another intermediate labeling, group 2ʹ, with the labeling of the glycerol backbone (half of 5 C–H is 2.5) in addition to the labeling of galactose and a fatty acyl chain in group 2. Group 2ʹ would have 20.5 and 23 D-labeling for MGDG 36:6 and DGDG 36:6, respectively. Observed group 2 in backward labeling might be a mixture of groups 2 (galactose and a FA labeling) and 2ʹ (glycerol, galactose and a FA labeling). The presence of group 2ʹ is attributed to a large pool of both labeled and unlabeled FAs in intermediate tissues, which can be randomly added to *sn*-1 and *sn*-2 positions.

Third, the backward labeling also revealed group 3ʹ that contains the recycling of unlabeled glycerol in the biosynthesis of new lipids. The non-binomial distribution of group 3 in the backward labeling (**Fig. 6**) is not surprising as the maximum number of removable D is limited, but it has a broader distribution than natural isotope distribution in fully H-labeled galactolipids. For example, MGDG 36:6 has the natural isotope abundance of 100:51:9 for M0:M1:M2 as seen in **Fig. 1C** or **Fig. S1**, but group 3 in **Fig. 6A** has much higher M1 and M2 as well as significant signals for M3–M6. This suggests that newly synthesized group 3 is not purely H-labeled. Here, they are referred to as group 3ʹ, as illustrated in **Fig. 7** with only glycerol unlabeled. Similar labeling is expected in forward labeling but not observed due to the very broad nature of group 3. It is odd to detect unlabeled (or pre-existing D-labeled in the backward labeling) glycerol in new tissues, but we speculate that they might have come from parent fronds where they were reserved as starch or other forms of oligosaccharides (Dörmann and Benning 1998).

### D_2_O stress and ^13^C-labeling

Despite its usefulness as a global isotope labeling strategy, *in vivo* D_2_O labeling has a critical downside due to its toxicity (Evans and Shah 2015). While the use of low D_2_O concentration can minimize adverse effects, we have chosen to use the maximum D_2_O concentration, 50%, which is possible without supplementation. It was an inevitable choice to maximize the separation of intermediate isotopologue groups. For example, a significant overlap is expected among the isotopologue groups with the 25% D_2_O concentration as simulated in **Fig. S8**. Another benefit of using the 50% D_2_O concentration is the symmetric isotopologue distributions, making the data interpretation easier. Although *L. minor* could propagate indefinitely in this high D_2_O concentration, D_2_O-induced stress is apparent as observed in the slow growth rate and smaller frond sizes (**Fig. 1A, B**), similar to that reported by others (Cope et al. 1965, Cooke et al. 1979, Evans et al. 2019). Evans et al. suggested that the growth inhibition by high D_2_O concentration might be due to the impacts on multiple metabolic pathways (Evans et al. 2019). We did not perform detailed physicochemical analysis of *L. minor* grown in the 50% D_2_O medium because it was thoroughly performed previously by Evans et al. (2019). When compared to *L. minor* grown in normal media, they found no obvious abnormalities in cellular morphology, a slight decrease in the degree of polymerization in cellulose (15%) and similar average weight (0.031 g vs 0.027 g for 35 fronds). Notable differences they reported include the slower growth rate (40%), lower chlorophyll content (0.065% vs 0.113% of total weight) and lower lignin content (8% vs 18% of total weight). A significant deuterium substitution, 40–50%, is found in the solid-state NMR analysis, corresponding to 80–100% of D-labeling efficiency.

This D_2_O stress did not affect the presence of three distinct isotopologue groups as supported by the backward labeling and ^13^C-labeling; however, their temporal changes are affected by about 2 d of delay according to the backward labeling experiment (**Fig. 6**). Cooke et al. observed a rapid loss of soluble protein and an increase in free amino acids when *L. minor* were transferred to 50% D_2_O. It was recovered in 2 d, but the protein synthesis was still inhibited by 20%, which would consequently lead to slow growth (Cooke et al. 1979). This is consistent with the result of our forward labeling experiment, in which the growth delay might be occurring only initially to adjust to the new stressed environment. Another downside of D_2_O labeling is the wide binomial distribution. This is because the use of the 50% D_2_O concentration produces half and half chance of hydrogen vs deuterium-labeling for every carbon-bound hydrogen that is being synthesized if we ignore the kinetic isotope effect, resulting in a symmetric Gaussian-like distribution. If it were possible to use 99% D_2_O, an almost complete D-labeling would have been achieved with a much narrower isotopologue distribution that can reveal more details of *in vivo* isotope labeling of each building block. Similarly, ∼90% ^13^CO_2_-labeling resulted in a much narrower isotopologue distribution than in 50% D_2_O labeling (**Fig. 4**).

Considering the wide isotopologue distributions and D_2_O-induced stress, ^13^CO_2_-labeling might be more effective than D_2_O labeling. As our preliminary experiment demonstrated, it can separate not only the three isotopologue groups but also glycerol backbone labeling of groups 1ʹ and 2ʹ that were not observed in D_2_O labeling. It was not possible, however, to perform almost complete (e.g. 99%) ^13^CO_2_-labeling in this preliminary experiment because of the need for a completely air-tight system. Such systems have been previously demonstrated, but it often requires many years of efforts to build an air-tight growth chamber with precise environmental control including ^13^CO_2_ concentration (Peters et al. 2018, Ćeranić et al. 2020). We were able to achieve an average of ∼90% ^13^C-labeling when compared to a simulation, but a broad distribution was unavoidable for groups 2 and 3 as the ^13^CO_2_ vs ^12^CO_2_ concentration was continuously changing. Nevertheless, carbon metabolism is not the same as hydrogens. Carbons in ^13^CO_2_-labeling enter the metabolic pathway of a plant system only through the Calvin cycle (Leegood 2013), but hydrogens in D_2_O-labeling can enter the metabolic pathway through multiple different pathways. As a result, the carbon flux and hydrogen flux would not be the same even when monitoring the same metabolic pathway. A careful comparison of mixed *in vivo* labeling, e.g. ^13^CO_2_ + H_2_O, ^12^CO_2_ + D_2_O and ^13^CO_2_ + D_2_O, may reveal some differences between the two fluxes.

### Metabolite flux information

The ultimate goal of MSI with *in vivo* isotope labeling would be to obtain the imaging of metabolite flux or tissue-specific/cell-specific flux information. Recently, Wang and coworkers illustrated an example of localization-specific flux information using MSI of rat kidneys with *in vivo* isotope tracing (Wang et al. 2022). They have monitored glycolysis metabolism by measuring M6 UDP-glucose while infusing [U-^13^C]-glucose and gluconeogenesis metabolism by measuring M3 UDP-glucose while infusing [U-^13^C]-glycerol. MSI data revealed high glycolysis activity in the medulla of rat kidney but high gluconeogenesis activity in the cortex. Similarly, in the current study, MSI with D_2_O labeling of *L. minor* revealed that there is no group 2 or 3 in old parent tissues, meaning that new FA biosynthesis does not occur in mature tissues or at least not involved in galactolipid biosynthesis. This is despite the fact that lipid restructuring is still being made in old tissues by recycling unlabeled “old” FAs evidenced by the presence of group 1ʹ.

Assuming that the mature plant tissues moved to the 50% D_2_O medium were in the pseudo-metabolic steady state because they almost stopped growing (see **Supporting Video S1** for example), unlabeled and group 1 of MGDG 36:6 are plotted between the two over time to provide flux information. These two groups are present only in old parent tissues unlike other isotopologue groups. As shown in **Fig. S9A**, **C** for MALDI-MS and ESI-MS data, respectively, the relative abundance of the labeled MGDG (group 1) increases exponentially with the asymptotic value of 28–35%, while a majority, 65–72%, of pre-existing (unlabeled) MGDG in old tissues does not further metabolize. Similar exponential changes are observed for DGDG 36:6 (**Fig. S9B, D**) but only 36–42% of the pre-existing (unlabeled) DGDG is present in old tissues at the asymptote. From ESI-MS data, the influx is estimated to be ∼22% per day and ∼30% per day for the synthesis of MGDG 36:6 and DGDG 36:6, respectively, assuming *t*_1/2_ = [Pool size]/*F*_in_ × ln(2) (Jang et al. 2018). This is only a rough estimation of the daily rate of synthesis and incorporation of labeled galactose into the lipid pools in the D_2_O stress environment.

### Limitations of this study

Despite its success, the current study has many limitations. Most importantly, D_2_O stress might have induced some differences from unstressed normal physiological conditions. While most qualitative observations are expected to be similar, some quantitative information may not be the same. One confirmed example is 2 d of delay in forward labeling compared to backward labeling (**Fig. 6**). The estimated influx of 22% and 30% per day for MGDG 36:6 and DGDG 36:6 syntheses in the final galactosylation step (group 1) might also be slightly higher in the unstressed condition (H_2_O media). There is a technical difficulty or consideration in this experiment coming from the effect of environmental humidity in the D_2_O concentration. A simple *in vivo* D_2_O labeling system adopted in this study using a Petri dish was exposed to ambient air for continuous gas exchange. Although the lid was almost closed to minimize water vapor exchange, some exchange was inevitable during the multiple days of plant growth. As a result, the D_2_O concentration decreased over time and subject to the laboratory humidity as shown in **Fig. S10**. At 70% laboratory humidity, 50 mol% of initial D_2_O concentration is decreased to ∼30 mol% in about a week, whereas 50 mol% of initial D_2_O concentration is decreased to only about ∼46 mol% at 25% humidity. The peak positions of each isotopologue group were greatly affected by the change in the D_2_O concentration, but the peak area seemed to be rather insensitive. Most of the experiments used in this study were obtained when the laboratory humidity was relatively low. When necessary, experiments were performed inside an environmental chamber to control the humidity.

### Future outlook

The current study exemplified some potentials of MSI*i* to elucidate the spatio-temporal details of galactolipid biosynthesis in duckweeds. We expect that there will be a lot more that this technology can offer to better understand plant metabolic biology. Some limitations, technical and biological, have been identified, but they could be overcome with improvements or with the help of other alternative approaches in parallel. For example, phospholipids, key intermediates of galactolipid biosynthesis, were not detected in the current experimental set-up. According to the preliminary thin layer chromatography (TLC)-ESI-MS analysis, their isotopologue analysis turned out to be very complicate and almost impossible with the current instrumentation because of overlapping isotopologues with multiple unsaturation. Future study will include additional method development and ultrahigh-resolution MS analysis for MSI*i* of phospholipids. Despite its advantage as a global labeling agent, D_2_O labeling has several limitations such as D_2_O-induced stress and broad isotopologue distributions. Other isotope labeling has their own pros and cons; ^13^CO_2_-labeling is also attractive in the study of plant biology, but technically challenging due to the need of gas tight growth chamber. In the future, we will build a relatively simple system for the ^13^CO_2_-labeling of duckweeds that can achieve an ∼99% ^13^CO_2_ concentration while controlling its concentration with the CO_2_ sensor. It may need the scrubbing of volatile organic compounds for a long-term plant growth (Peters et al. 2018) but may not be necessary for a short-term experiment. However, ^13^CO_2_-labeling may not be completely compatible with D-labeling as it is carbon-centric. The use of organic precursors, e.g. [U-^13^C]-glucose or [1,2]-^13^C_2_-glucose, is another option for ^13^C-labeling, which can also specifically define the reaction pathways that are being studied while limiting the scope of the study (Lee et al. 1998, Dellero et al. 2024).

Some technological limitations in MSI*i* are coming from the limitations in MS. Ultrahigh mass resolution is necessary to separate a complex mixture of various isotope labeling. For example, to distinguish the same two lipids with ^13^C_1_ vs D_1_ labeling (2.9 mDa difference; e.g. ^13^C_1_-MGDG 36:6 vs D_1_-MGDG 36:6) requires the mass resolution to be higher than 280,000, which is not obtainable with the Orbitrap used in this study. It will be possible but challenging even with the most advanced MS technology such as Fourier Transform Ion Cyclotron Resolution (FTICR) and would not be routinely available to many researchers. Furthermore, a trapping type of mass spectrometers such as Orbitrap and FTICR has isotope abundance errors due to the space charge effect when storing ions for a long period to obtain higher mass resolution (Hohenester et al. 2020). As a result, one needs to be very cautious in data analysis, not to overinterpret the result and clarify the quantitative limitations. Some fundamental study would be necessary to define the limitations and find practical alternatives. Regardless, MSI*i* is a newly emerging technology and is expected to provide insights into metabolic biology that has not been previously available. Potential applications include MSI*i* of crop seeds under stress conditions or with genetic engineering, which may provide further insights into the spatio-temporal change in lipid biosynthesis and provide solutions for overcoming the current bottleneck in genetic engineering.

## Materials and Methods

### Chemicals

Liquid Chromatography/Mass Spectrometry-grade isopropanol, chloroform, methanol and water, sodium acetate and potassium acetate and lactic acid were obtained from Fisher Scientific (Hampton, NH, USA). Isotope products, 70 or 99.9% D_2_O and 99.8% Ba^13^CO_3_, were purchased from Cambridge Isotope Laboratories, Inc. (Tewksbury, MA, USA). MS-grade TLC from Sigma-Aldrich (St. Louis, MS, USA) was used for lipid separation. CO_2_-free air Airgas (Radnor, PA, USA) was used to flush out ^12^CO_2_ in ambient air.

### Plant materials

*Lemna minor* plant was originally obtained from VWR (catalog number 470194-692) and propagated in 0.5× SH (Phyto Technology Laboratories, Lenexa, KS, USA) for many months on a laboratory bench. Plant media and growing beakers were sterilized by autoclaving under a liquid cycle. A cool white LED shop light was used as a light source with the photon density of 60 µmol m^−2^ s^−1^ and the light cycle of 16 h/8 h. The ambient conditions were largely relying on the building air circulation system. The laboratory temperature was typically at 20–25ºC, and the ambient humidity varied between 20 and 35% in winter and 55 and 70% in summer. The medium is regularly replaced every ∼7 d.

### D_2_O labeling experiment

For the forward D_2_O-labeling experiment, 15–20 healthy fronds grown in control media were transferred into each of six small Petri dishes with 14 ml of 50% D_2_O, H_2_O:D_2_O (50:50, mol/mol) and 0.5× SH media and grown for different periods, 1, 2, 3, 5, 10 and 15 d. For the backward labeling, three replicates of 15–20 plants grown in 50% D_2_O for 3 months were transferred to each Petri dish of H_2_O media.

It should be noted that high room humidity, especially on rainy summer days, could greatly affect D-labeling (**Fig. S10**). Both forward and backward labeling experiments were performed at low humidity (∼25%), and its effect on D-labeling is expected to be minimal, an average of ∼2% for the 5-day labeling data. The D_2_O concentration of media was measured using infrared spectroscopy, Bruker Tensor 37 FTIR. An environmental chamber (ICH110L; Memmert, Schwabach, Germany) was used to control humidity for the forward labeling experiment in **Fig. 6**, in which temperature and humidity were controlled at 20°C and 25%, respectively.

### ^13^CO_2_ labeling experiment

An Erlenmeyer flask (250 ml) with a three-hole screw cap was set up and connected with CO_2_-free air and lactic acid–containing syringe as shown in **Fig. S6**. The third hole is blocked with an end cap, which was used to release or seal the air inside. The flask contained two 10-ml beakers, one for 5–7 plants in 5 ml of 0.5× SH medium and the other for ∼1 mg of Ba^13^CO_3_ powder. At first, CO_2_-free air was introduced into the flask to flush out ambient ^12^CO_2_ gas inside the flask. After 2 min, the cylinder valve and the end cap were closed almost simultaneously and then ∼60 µl of lactic acid (3.3%, w/v) was introduced into the Ba^13^CO_3_ beaker to produce ^13^CO_2_ (Ida and Kudo 2008). As the system was not perfectly sealed and there might be a buildup of organic gases over time, this process was repeated every 3 h to flush out the air and freshly produce ^13^CO_2_ by adding lactic acid during 16 h of daylight for 0–3 d.

### MALDI-MS imaging

For MALDI-MSI samples, three healthy individual plants were selected at each time point. The fracturing method was employed to expose the middle layer of the frond as described elsewhere (Klein et al. 2015). Briefly, the plants were attached to a packing tape, vacuum dried, enclosed with the tape to also attach the other side of fronds with the tape, passed through a rolling mill to make a mechanical damage to the internal tissues and then pulled over two ends of the tape piece to produce two split half-fronds exposing the internal mesophyll layers. The top half layer with the adaxial side attached to the tape was used for the MALDI-MSI. The tissue samples were sputtered with gold for 20 s at 40 mA (Cressington 108; Ted Pella, Redding, CA, USA) to provide an electrically conductive surface and then sprayed with 2,5-dihydroxybenzoic acid using a TM sprayer (HTX Technologies, Chapel Hill, NC, USA).

An Orbitrap mass spectrometer Q Exactive HF (Thermo Scientific, San Jose, CA, USA) equipped with a medium pressure (∼8 Torr) MALDI source (Spectroglyph, Kennewick, WA, USA) was used in this study. A 349-nm Neodymium-doped yttrium lithium fluoride (Nd:YLF) laser (Explorer One, Spectra Physics, Milpitas, CA, USA) was used with the laser energy of ∼1 µJ per pulse and the spot size of 15 µm. The MALDI-MSI dataset acquired for an *m*/*z* value of 100–1200 in positive mode with a raster step of 30 µm and the mass resolution of 120,000 at *m*/*z* 200. Data were processed with MSiReader (Robichaud et al. 2013) to generate MS images, Xcalibur (Thermo) to extract raw data, in-house Python code to filter isotopic peaks, and MATLAB to deconvolute group 2 vs group 3 isotopologue distributions.

### ESI-MS analysis

Lipid extraction followed by ESI-MS analysis was used for the isotopologue group comparison between parent vs daughter fronds (**Fig. S3**) and the backward labeling experiment (**Fig. 6**). The lipid extract method was derived from the single-extraction method by Shiva et al. (2018). Harvested *L. minor* plants were gently dried with Kimwipes, incubated in preheated 75°C solution of 0.01% BHT isopropanol (w/v) for 15 min and cooled to room temperature. Chloroform, methanol and water were added to make the final solvent mixture with isopropanol:chloroform:methanol:water in the ratio of 31/28/38/3 (v/v/v/v). The extract was shaken at ∼500 rpm for 12 h. The parent vs daughter frond experiment was performed by directly infusing the lipid extract into the Orbitrap MS with ESI using a sample loop after adding potassium acetate solution to a final concentration of 2 mM potassium.

For the backward labeling experiment, TLC was employed to separate lipid species. The lipid extracts from both forward and backward labeling sample sets were dried under nitrogen and resuspended with 0.16 volume of chloroform:methanol (2/1, v/v) mixture. Approximately 10 µl of condensed lipid extract was deposited onto a TLC plate predeveloped with chloroform:methanol (1/1, v/v) to remove impurities. A mixture of chloroform:methanol (72/28, v/v) was used as the developing solvent (Hölzl and Dörmann 2021). After separation, lipid bands of MGDG and DGDG were scraped off, dissolved in the mixture of chloroform:methanol (2/1, v/v) and gently shaken for 15 min. When silica gel was settled at the bottom of the test tubes, the transparent solution was taken and sodium acetate solution was added to a 2 mM final concentration and then injected to the ESI loop. All lipid data were acquired in the *m*/*z* range of 600–1100 with positive mode and the resolution of 240,000 at an *m*/*z* value of 200.

## Supplementary Material

pcae032_Supp

## Data Availability

MSI data used in this study are available upon request to the corresponding author.
