# Correction to: Spatio-Temporal Study of Galactolipid Biosynthesis in Duckweed Using Mass Spectrometry Imaging and *in vivo* Isotope Labeling

**DOI:** 10.1093/pcp/pcae064

**Published:** 2024-06-15

This is a correction to:

Vy T Tat, Young Jin Lee, Spatio-Temporal Study of Galactolipid Biosynthesis in Duckweed Using Mass Spectrometry Imaging and *in vivo* Isotope Labeling, *Plant and Cell Physiology*, 2024; pcae032, https://doi.org/10.1093/pcp/pcae032

The original published version of this article contained an incorrect grant number; the funding information has now been corrected in the available online version, from:

‘National Science Foundation, Plant Genome Research Program (GR-025234-00001)’.

To:

‘National Science Foundation, Plant Genome Research Program (2150468)’.

