# Supplementary material for: Spatio-Temporal Study of Galactolipid Biosynthesis in Duckweed Using Mass Spectrometry Imaging and in vivo Isotope Labeling
Source: Plant Cell Physiol. 2024 Mar 28;65(6):986–98. doi: 10.1093/pcp/pcae032 (PMC11758584; doi:10.1093/pcp/pcae032)
Supplement: pcae032_Supp [file pcae032_supp.zip › suppl_data/pcp-2024-e-00008-File009.pdf]

Supporting information for:
Spatiotemporal Study of Galactolipid Biosynthesis in Duckweed with Mass Spectrometry Imaging and *in vivo* Isotope Labeling

Vy T. Tat and Young Jin Lee*

Department of Chemistry, Iowa State University, Ames, IA 50011, United States

*Corresponding author: Young Jin Lee. Email: yjlee@iastate.edu

**Table of Contents**	
Supporting Methods (Pg 1)	
Removal of natural isotope abundances in isotopologue distributions	Pg 1
Supporting Figures (Pg 2-6)	
Figure S1. Deconvolution of natural isotope abundances	Pg 2
Figure S2. D-labeling isotopologue distribution simulation for MGDG 36:6	Pg 3
Figure S3. ESI-MS analysis of galactolipid extracted from parent and daughter fronds.	Pg 3
Figure S4. Isotopologue distribution of Group 1 in DGDG 36:6	Pg 4
Figure S5. Isotopologue distribution of pheophytin *a*	Pg 4
Figure S6. $^{13}CO_2$ labeling experiment setup.	Pg 5
Figure S7. ^{13}C-labeling isotopologue distribution simulation for MGDG 36:6.	Pg 5
Figure S8. D-labeling isotopologue distribution simulation for MGDG 36:6 with p of 0.25	Pg 6
Figure S9. Relative abundance change of unlabeled and Group 1.	Pg 6
Figure S10. The change of D_2O concentration over time by room humidity.	Pg 7

Supporting Methods

Removal of natural isotope abundances in isotopologue distributions

The high-resolution mass spectrometer used in this study can distinguish the lipids of our interest and their deuterated peaks from almost all interferences; however, it does not have sufficient mass resolution to separate ^{13}C- or other natural isotopes from deuterated peaks. For example, $^{13}C_1$-MGDG 36:6 has the mass difference of only 2.9 mDa from D_1-MGDG 36:6. As a result, natural isotopes will appear as combined with deuterated peaks in our mass spectra. To remove the contribution of natural isotope abundance in deuterium isotopologue distribution by *in vivo* labeling, we have performed a deconvolution as described below with one-day labeled MGDG 36:6 as an example. The main assumption in this process is the natural isotope abundance obtained from the control sample would be the same for each deuterated peak. Due to the experimental measurement error, this assumption is not exactly valid, and some subtraction error is expected.

First, the monoisotope and isotope peaks are extracted from the mass spectra of control and deuterated samples. The peak abundances are normalized to have the monoisotope peak as 100 (**Figure S1A** and **S1B**). Natural isotopes are considered up to M+2 assuming M+1 is mostly composed of $^{13}C_1$ isotope and M+2 is mostly composed of $^{13}C_2$, $^{18}O_1$, and $^{41}K_1$ isotope (if potassiated). Second, natural isotope abundances obtained from control sample are subtracted from the deuterated sample (**Figure S1C**). The leftover M+1 abundance is attributed to the labeling of one deuterium, and its natural isotope abundance is expected to contribute to M+2 and M+3 peaks with the same ratio of control as illustrated in **Figure S1C**. Third, as a subsequent step, 50.52% and 8.56% of M+1 peak is subtracted from M+2 and M+3 peaks, respectively, and the leftover M+2 peak is considered as the labeling of two deuterium. As we continue subtracting natural isotope abundance and assigning leftover peaks as deuterated, the isotopologue distribution can be obtained as shown in **Figure S1D**.

This process is not perfect due to the experimental errors, which seems especially high for D_1 and D_2-labeling in early days of labeling when $^{13}C_1$ or $^{13}C_2$ contributions are high. When there is no monoisotope peak (e.g., Day 15), normalization was made to the first deuterated peak, and the same procedure was followed. It should be noted that this process of natural isotope abundance removal makes only meaningful difference for low number of deuterations and was not performed for the backward labeling nor ^{13}C-labeling to avoid confusion.

Supporting Figures

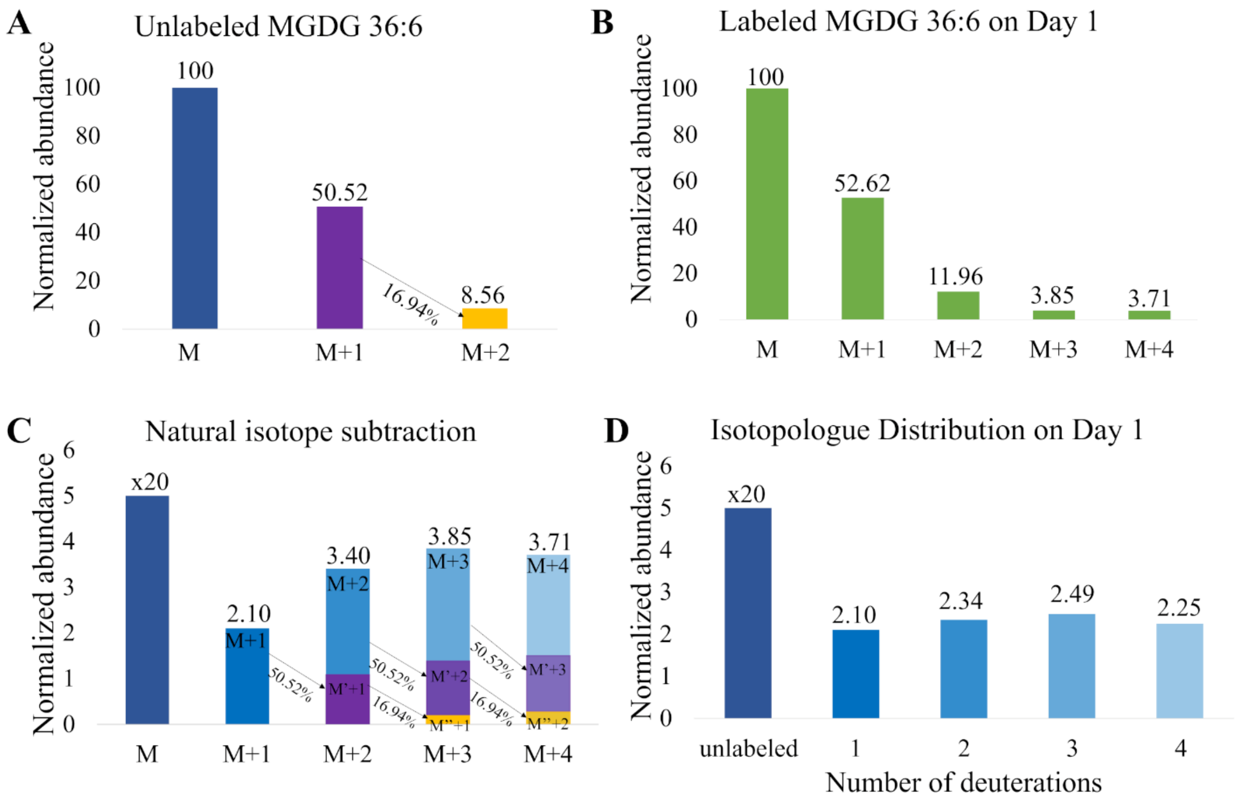

Figure S1. **Illustration of the deconvolution process to remove natural isotope abundance and obtain D-labeling isotopologue distribution.** MGDG 36:6 data was shown as an example after *L. minor* was moved to 50% D_2O medium for one day. **(A)** Normalized natural isotope abundance from the control sample. **(B)** Normalized isotope abundance for one-day labeled sample shown up to M+4. **(C)** Natural isotope abundances originated from the unlabeled monoisotope were subtracted from the labeled sample at M+1 and M+2. Also illustrated are the contribution of M+1 and M+2 natural isotope abundances from each deuterated peak to the subsequent isotope peaks. **(D)** Isotopologue distribution of D-labeling on Day 1 after the removal of natural isotopes.

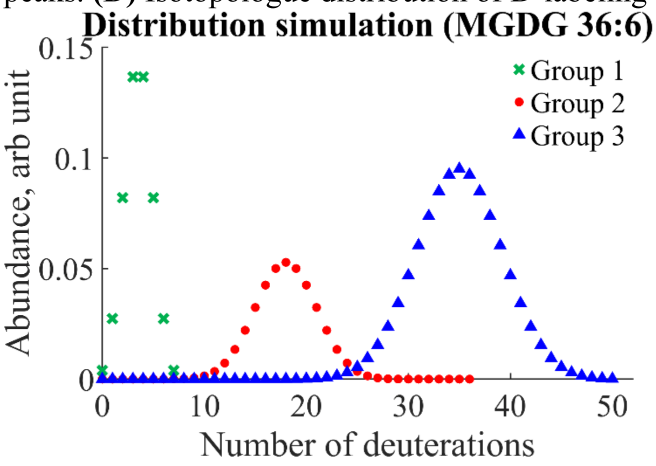

Figure S2. D-labeling isotopologue distribution simulation for MGDG 36:6 using binomial function. Excel function, BINOM.DIST, is used with p (D_2O concentration) of 0.5 and n (number of C-H) of 7, 36, and 70 for Group 1, 2, and 3, respectively. Relative abundances are arbitrarily adjusted to make it similar to **Figure 2A** by multiplying 0.5, 0.4, and 1, respectively.

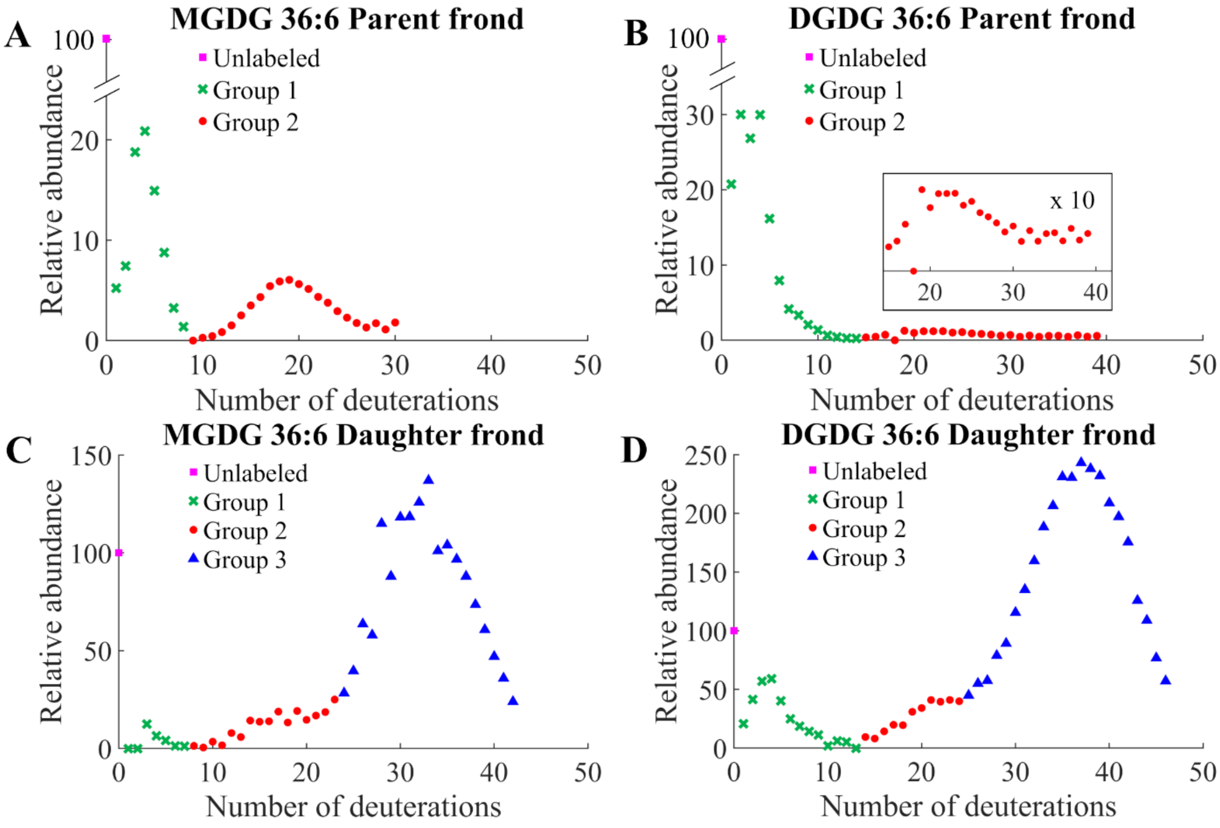

Figure S3. ESI-MS analysis of (A, C) MGDG 36:6 and (B, D) DGDG 36:6 for the lipids extracted from **(A, B)** parent fronds and **(C, D)** daughter fronds of *L. minor* grown in 50% D_2O for five days. Natural isotope abundance is subtracted.

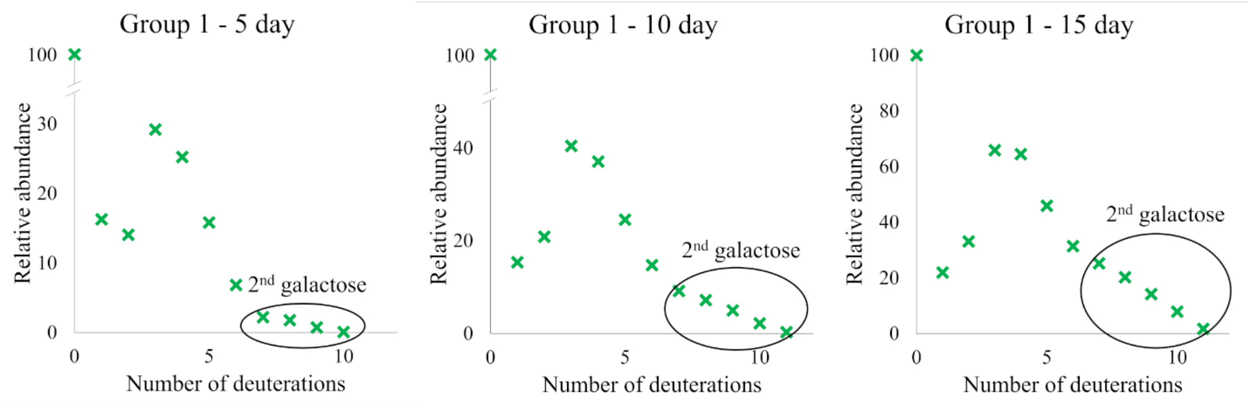

Figure S4. **Isotopologue distribution of Group 1 in DGDG 36:6** after 5, 10, and 15 days in 50% D_2O medium. The 2nd galactose labeling contributes to the shoulder of the 1st galactose isotopologue distribution.

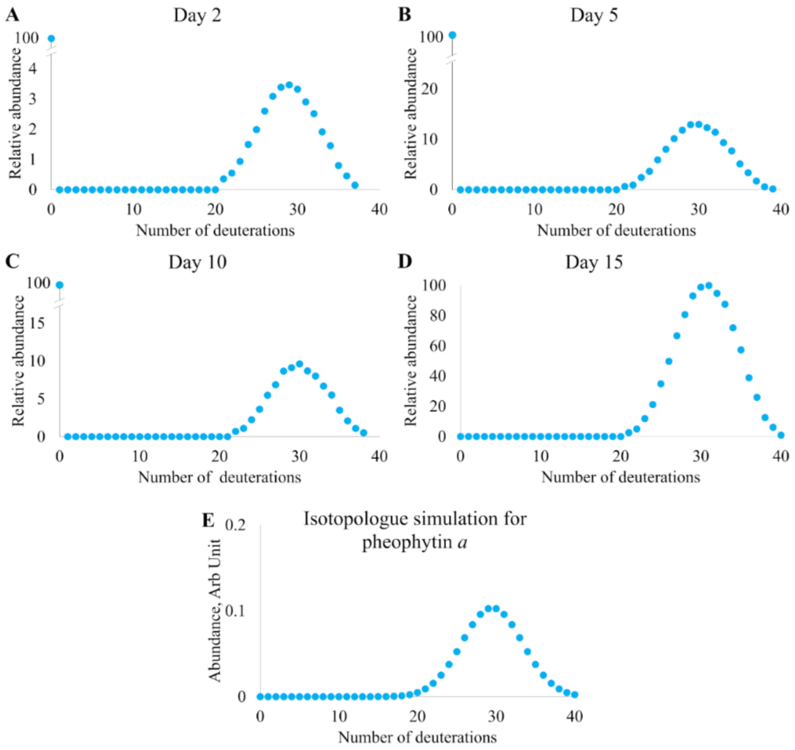

Figure S5. **Isotopologue distribution of pheophytin *a*** from MALDI-MS of *L. minor* after grown in 50% D_2O for **(A)** 2, **(B)** 5, **(C)** 10, **(D)** 15 days, compared to **(E)** the simulated pheophytin *a* with the binomial distribution of p = 0.5 and n = 59.

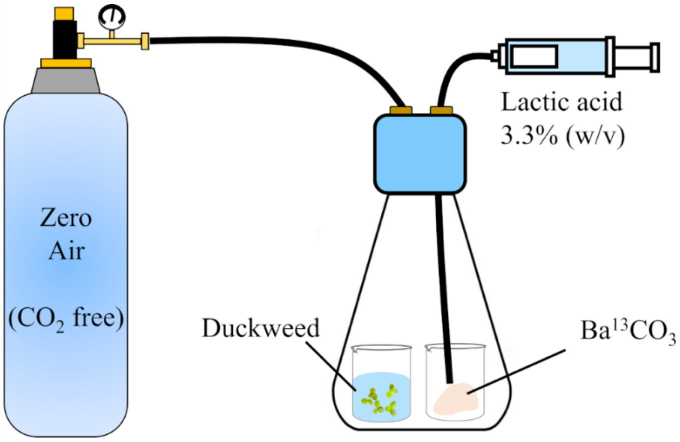

Figure S6. $^{13}CO_2$ labeling experiment setup.

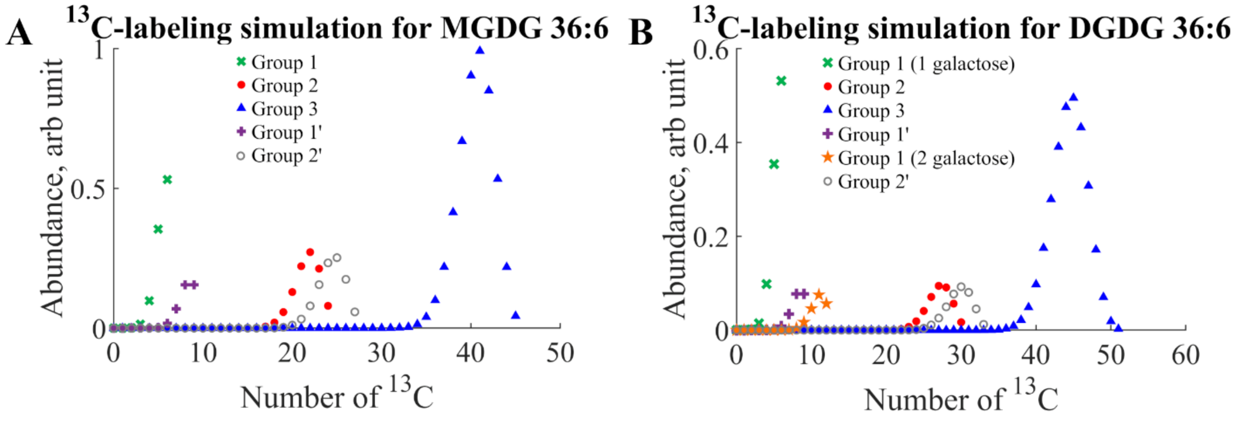

Figure S7. **Simulation for ^{13}C-labeling isotopologue distribution**. **(A)** Binomial distributions for MGDG 36:6 are calculated using p = 0.9 and n = 6, 9, 24, 27, 45 for Group 1, 1', 2, 2', 3, respectively. **(B)** Binomial distributions for DGDG 36:6 are calculated using p = 0.9 and n = 6, 9, 12, 30, 33, 51 for Group 1 (1 galactose), 1', 1 (2 galactose), 2, 2', 3, respectively. Abundances are arbitrarily adjusted to make them similar to the experimental distributions.

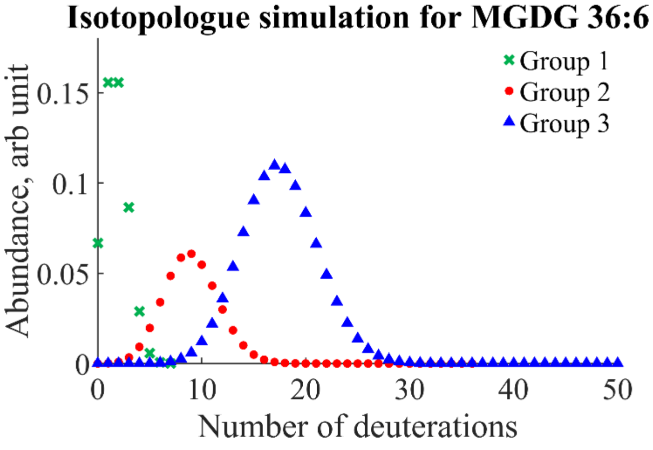

Figure S8. D-labeling isotopologue distribution simulation for MGDG 36:6 with p of 0.25. Binomial distribution was simulated using p of 0.25 and n of 7, 36, and 70 for Group 1, 2, and 3, respectively. Abundances are arbitrarily adjusted to make them similar to Figure 2A by multiplying 0.5, 0.4, and 1, respectively.

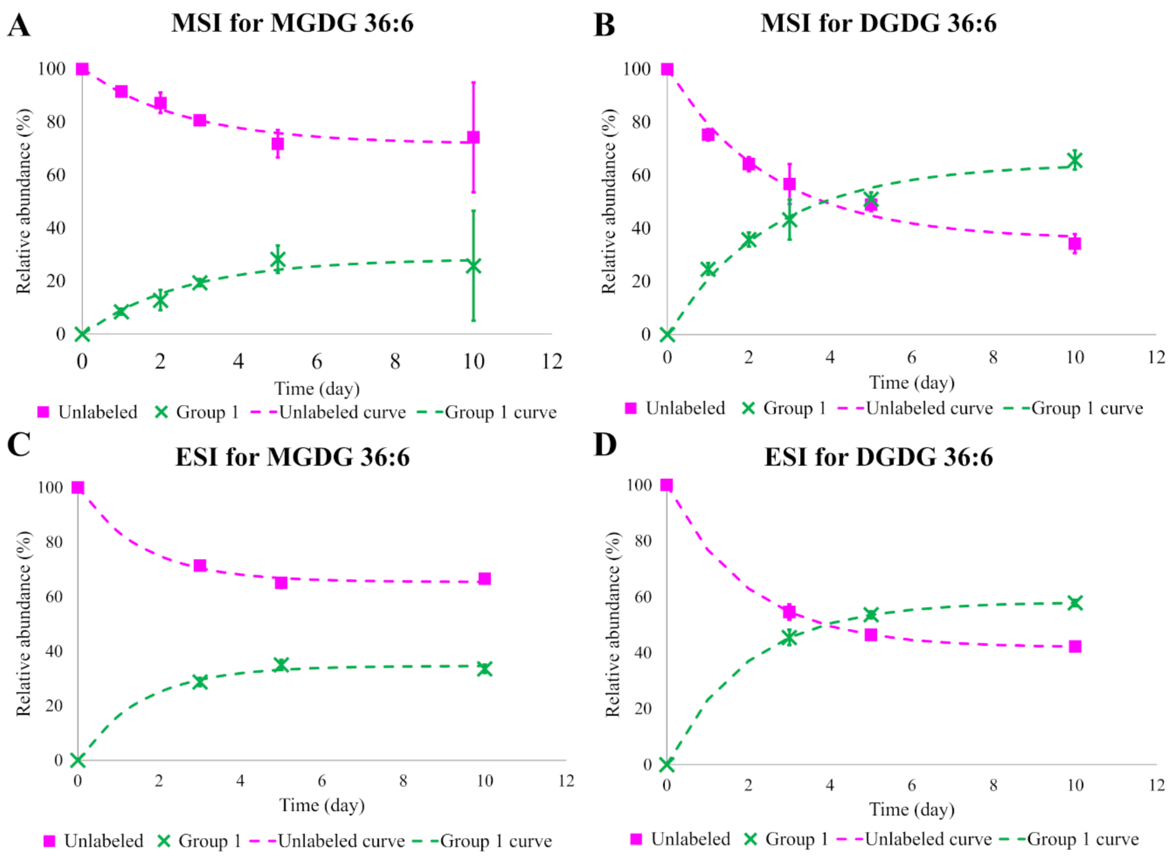

Figure S9. **Relative abundance changes of unlabeled vs Group 1 for MGDG 36:6 (A, C) and DGDG 36:6 (B, D) obtained from MALDI-MSI (A, B) and TLC-ESI-MS (C, D).** MALDI-MS data are replotted from Figure 2 by normalizing the sum of the two as they are present only in old tissues. Data on Day 15 is not included as the parent frond is dying out by then and separated from the daughter fronds. Exponential fitting was made using 0-10 Day data.

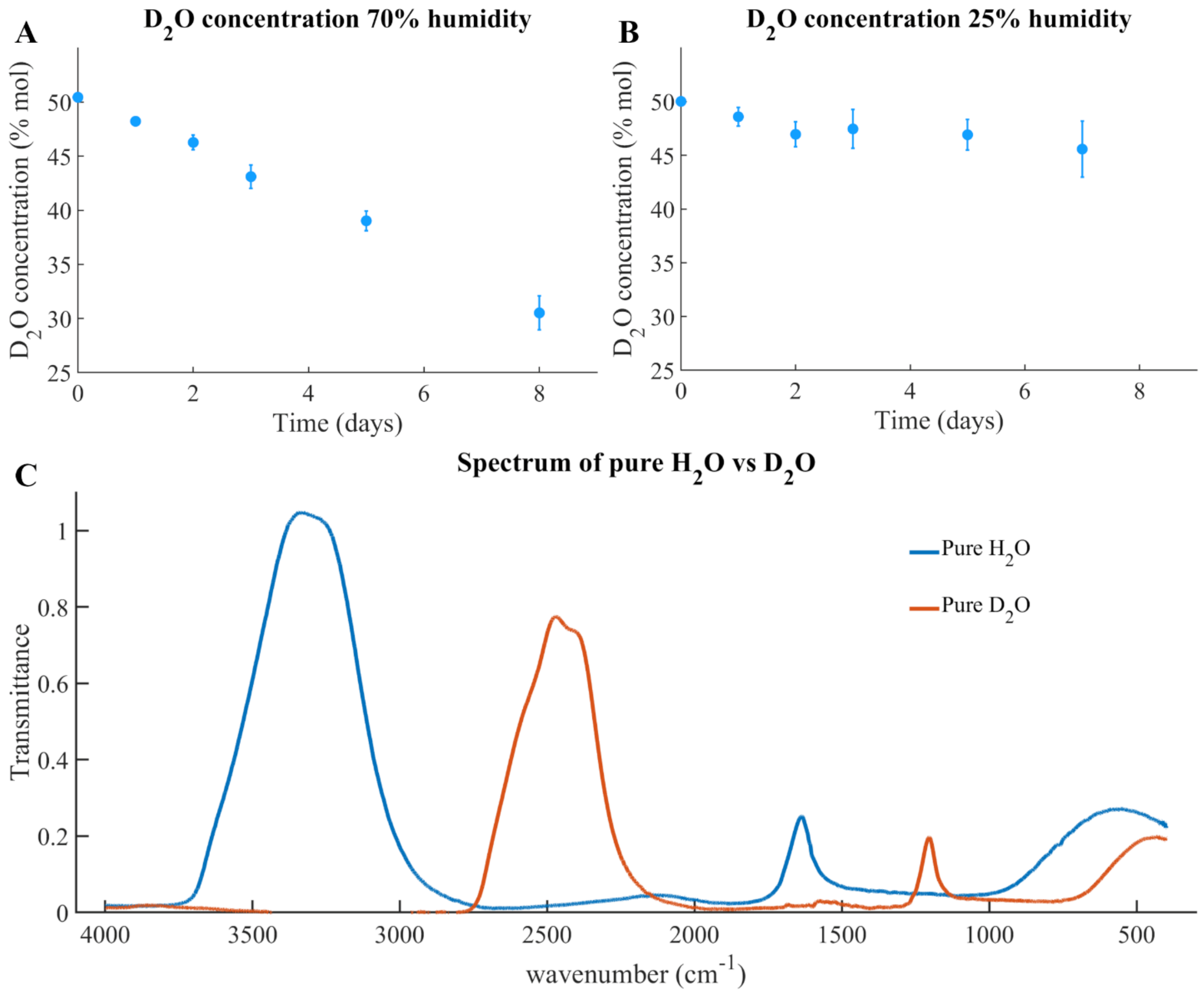

Figure S10. The change of D_2O concentration over time in **(A)** 70% and **(B)** 25% ambient humidity. **(C)** ATR FT-IR was used for this measurement using OH (3400 cm^{-1}) and OD (2500 cm^{-1}) stretch vibration. The ratio of OD peak area vs the sum of OD and OH peak area was used to calculate the D_2O concentration after correcting molar absorptivity between the two.
